# Word Processing differences between dyslexic and control children

**DOI:** 10.1186/1471-244X-6-5

**Published:** 2006-01-27

**Authors:** Isabella Paul, Christof Bott, Christian Wienbruch, Thomas R Elbert

**Affiliations:** 1 Department of Psychology, University of Konstanz, Germany, PO-Box D 25, 78457 Konstanz, Germany

## Abstract

**Background:**

The aim of this study was to investigate brain responses triggered by different wordclasses in dyslexic and control children. The majority of dyslexic children have difficulties to phonologically assemble a word from sublexical parts following grapheme-to-phoneme correspondences. Therefore, we hypothesised that dyslexic children should mainly differ from controls processing low frequent words that are unfamiliar to the reader.

**Methods:**

We presented different wordclasses (high and low frequent words, pseudowords) in a rapid serial visual word (RSVP) design and performed wavelet analysis on the evoked activity.

**Results:**

Dyslexic children had lower evoked power amplitudes and a higher spectral frequency for low frequent words compared to control children. No group differences were found for high frequent words and pseudowords. Control children had higher evoked power amplitudes and a lower spectral frequency for low frequent words compared to high frequent words and pseudowords. This pattern was not present in the dyslexic group.

**Conclusion:**

Dyslexic children differed from control children only in their brain responses to low frequent words while showing no modulated brain activity in response to the three word types. This might support the hypothesis that dyslexic children are selectively impaired reading words that require sublexical processing. However, the lacking differences between word types raise the question if dyslexic children were able to process the words presented in rapid serial fashion in an adequate way. Therefore the present results should only be interpreted as evidence for a specific sublexical processing deficit with caution.

## Background

It is widely agreed that a core deficit of dyslexia is reduced phonological awareness (the reduced ability to discriminate speech-sounds in spoken words [1-6]. Phonological awareness is not only assumed to be a pre-requisite for speech-perception, but also for learning the correspondence between graphemes and phonemes which again is crucial for reading and writing [1,7-10]. According to dual route models of reading (e.g. [11]), the pronunciation of words can be retrieved in two different ways, depending on the frequency and regularity of a word. High frequent (HF) words are more familiar to the reader, as they appear more often in spoken and written language. It is likely that the visual forms of HF words are directly associated with their meaning in the same way as images are [12]. Therefore it is possible to read a word at a glance, if it is well-known enough. This way of reading is often termed "direct route" or "lexicosemantic route". In contrast, other strategies may be used to decode low frequent (LF) words or pseudowords (PS). If a word is not familiar to the reader, it is necessary to phonologically assemble the word from sublexical parts following grapheme-to-phoneme correspondences in order to read and retrieve the meaning correctly. This route is called "graphophonological route", "indirect route" or "sublexical route".

Due to their lack of phonological awareness, it appears reasoned that dyslexics should be most impaired reading novel or unfamiliar words, where successful decoding relies on the application of the graphophonological route. Castles and Coltheart [13] investigated the reading performance of 53 dyslexic and 56 age matched control children. Children were to read aloud regular, irregular and PS words. They found that the majority (72%) of the dyslexic children were below the confidence limit for pseudoword reading, i.e. they had difficulties reading via the graphophonological route. Only 19% of the dyslexic children were exclusively impaired reading irregular words with a control like performance reading PS words. I.e. these children were able to successfully apply grapheme-phoneme-conversion rules necessary for pseudoword reading while being impaired using the direct route.

Visual word processing differences between high and LF words have been revealed in several studies measuring electrical or magnetic brain activity. Sereno et al. [14] reported higher EEG amplitudes for LF words in comparison to HF words in anterior parietal and occipital regions between 132 and 164 ms. Assadollahi and Pulvermüller [15,16] found in an MEG study that LF words led to stronger brain responses than HF words starting as early as 120 ms post stimulus. Calculating source localisations of the effects, they found that the frequency effects were strongest over a left occipito-temporal area. Hauk and Pulvermüller [17] also reported higher EEG amplitudes for LF words in an early timeframe from 150–190 ms (most pronounced at left occipital electrodes), as well as in a later timeframe between 320 and 360 ms for parietal leads. Interestingly, Proverbio et al. [18] found similar P150 EEG amplitudes for both PS words and words when their lexical frequency was low. The authors concluded that highly familiar words are recognized as unitary objects at early processing stages, while this is not the case for unfamiliar words. Rudell [19] visually presented HF and LF words in the EEG using the rapid stream paradigm [20]. The component elicited using this paradigm is called recognition potential (RP). A target word (HF or LF) was presented for 200 ms and then immediately followed by three different letter-strings. Data were recorded at two occipital electrodes. They reported a frequency effect with HF words having a shorter RP peak latency than LF words (266 vs. 292 ms).

All these findings reflect distinct cortical processing of HF and LF words at relatively early processing stages (between 100 and 360 ms), possibly reflecting different reading strategies. Hauk and Pulvermüller [17] postulate that the synaptic connections representing a word become more and more efficient, the more often a word is encountered. As a consequence less activation is necessary to activate the corresponding word. Thus, it appears reasoned that the graphophonological route will draw more processing resources than the direct route.

Not many studies have investigated word reading varying word frequency in dyslexia. The studies that did, however, found rather unexpected results. Johannes et al. [21] visually presented HF and LF words to 6 dyslexic adults and control subjects while measuring EEG. Each word was presented twice. In both groups they found LF words to elicit higher N400 amplitudes than HF words. While control subjects showed an amplitude decrease for both word types in the repeat condition, this decrease was only present for LF words in dyslexics. The authors inferred that N400 amplitude for HF words is already reduced on the first encounter in dyslexics. Johannes and colleagues interpreted this as a result of enhanced semantic integration of HF words, having compensatory function. The finding is surprising since one would have expected to find differences between dyslexic and control subjects processing LF words (only LF words require graphophonological reading). It should be noted, however, that the sample size in this study was very small and the dyslexics were recruited at a university, implicating that they were well compensated dyslexics.

Rüsseler and colleagues [22] investigated recognition memory for HF and LF words in 12 adult dyslexics and 12 adult controls in the EEG. Again, no specific processing differences for LF words were found between the groups. Recognition memory for both HF and LF words was reduced in dyslexics. Additionally, an old/new effect for the P600 component (that was stronger for LF words) was only present for control subjects. The authors explained this by a reduced recognition memory in dyslexic subjects, regardless of word type. Like in the study by Johannes et al. [21], it should be mentioned, however, that the dyslexic sample consisted of highly compensated university students, thus raising the question if the results can be generalised.

Hyönä and Olson [23] examined eye fixation patterns of 21 dyslexic and 21 younger, reading-age matched control children during reading of HF and LF words. They did not find the two groups to differ in number and length of fixations. The more "difficult" LF words attracted more and longer fixations than HF words. The authors interpreted their finding as support for a maturational lag hypothesis in dyslexia, since performance of the younger control group (mean age 10 years) resembled performance of the older dyslexic group (mean age 14 years). However, reading correctness did differ between the groups. Dyslexic children made twice as many word substitution errors (e.g. *travelled *instead of *traversed*) and 50% more nonword substitution errors (e.g. *compendent *instead of *competent*) than control children, with 95% of the latter and 76% of the word substitutions occurring on LF words.

In summary, the lack of group differences for processing LF words might be a consequence of the dyslexic samples being highly compensated university students [21,22] or of the dependent measure (eye fixations) not being sensitive enough to mirror the pattern found in the behavioural data [23].

The aim of the present study was to investigate cortical processing of HF, LF, and PS words in a representative dyslexic sample and a matched control group. We were not interested in semantic processing of different word types, but in automatic processes triggered by a visual word stimulus – like the initiation of different reading strategies depending on the familiarity of the stimulus. Thus, we chose to visually present different word types in a rapid serial visual presentation (RSVP) design (1 stimulus per 350 ms) while measuring cortical activity in the MEG. Since processing differences between HF and LF words have been found on early components (as early as 120 ms) probably reflecting pre-semantic processes of different reading strategies, we thought it possible to detect such differences when faster presentation rates than 1 stimulus per 800 ms – 1 stimulus per 2000 ms are used. The assumption that early word processing differences can be found using higher presentation rates than the ones generally used in electrophysiological word reading studies is supported by the work of Rubin and Turano [24]. They visually presented words as a conventional text passage (PAGE) or in a rapid serial fashion (RSVP) and found that subjects were able to read (and comprehend) 1100 words/min (one word per 54 ms) when reading words in RSVP while only 300 words/min in the PAGE condition. The authors argue that saccadic eye-movements (being more prominent in the PAGE condition) impose an upper limit on reading speed.

We assumed RSVP to be an appropriate tool to investigate word processing in dyslexia, since it allows setting focus on early, more *automatic *aspects of word processing induced by visual word stimuli. Control and dyslexic subjects were not expected to differ processing HF words, since the majority of dyslexics is not impaired reading highly familiar words. We did expect to find group differences for low frequent words: Skilled readers should be able to successfully decode low frequent words, while dyslexic readers should have difficulties doing so (due to their reduced ability to apply grapheme-phoneme correspondences). No specific hypotheses were formulated for PS word processing. We chose to analyse the stimulus-evoked cortical activity by the means of wavelet transformation, since it offers additional information about the spectral frequency of the effects.

## Methods

### Generation of the sample and behavioural tests

The participating children were contacted through 14 primary schools in or around Konstanz, Germany, and attended either 3^rd ^or 4^th ^grade. Schools were asked to name children with massive problems reading and spelling, as well as children without any such difficulties. Both parents and children gave informed consent to participate in the study. In order to objectively classify the children to be dyslexic, all children underwent a test-battery that was designed to assess a variety of abilities ranging from spelling and reading to phonological abilities (*DRT *(Diagnostischer Rechtschreibtest 3^rd ^grade [25]; 4^th ^grade [26]): Standardized spelling test; *ZLT *(Zürcher Lesetest [27]): Standardized reading test; *SPM *(Standard Progressive Matrices, German version [28]): non-verbal IQ-test; Non-standardised *Word reading*: List of words with increasing difficulty to be read aloud; Non-standardised *Pseudoword reading*: List of pseudo-words with increasing difficulty to be read aloud; *Mottier-Test *[29]: Pseudowords with increasing difficulty are read aloud by the experimenter and are to be repeated by the child; *Dictation *[30]: only words were used that are spelled as one "hears" them, i.e. no knowledge about spelling rules or exceptions is necessary; *Categorical perception*: judgement, if a syllable sounds more than "ba" or "da", when the formant transition period of the syllable is varied on a ten-item continuum. Item 1 on the 10-item continuum (12 items per step) represents a clear /ba/, item 10 a clear /da/. Categorical perception performance is quantified by the formula  with a_i _representing the number of responses for /ba/ and b_i _the number of responses for /da/. A high categorical perception index indicates reliable and correct categorisation of /ba/ and /da/). If a child, who was classified as being dyslexic by the teacher, was not significantly worse than the norm-sample in the standardized spelling test, he/she was excluded from the study. Control children who performed significantly worse than the norm sample in the spelling test were either excluded or classified as dyslexic.

### Subjects

20 control children and 55 dyslexic children participated in the study. The two groups did not differ statistically in age (F(1,73) = 0.24; p = 0.6, range 8–10 years), handedness [31] (X^2^(1,73) = 1.3; p = 0.3) or gender distribution (X^2^(1,73) = 0.01; p = 0.9). Table 1 displays the group mean results of the behavioural tests.

**Table 1:** One way ANOVAs of dependent variables SPM, DRT (T-values); ZLT Correctness, Word Reading Correctness, Pseudoword Reading Correctness, Mottier Test, Dictation (% correct); Word Reading Time, Pseudoword Reading Time (seconds); ZLT Reading Time (seconds/no words); Categorical perception (Index) and GROUP (control, dyslexic) as between group factor.

	Control	Dyslexic	F (1,73)	p
SPM *T*	63	51.4	15.2	0.0002
DRT *T*	58.1	36.9	194.7	<0.0001
ZLT Correctness *%correct*	97	87	22.4	<0.0001
ZLT Reading time *seconds/no words*	0.67	1.45	22.5	<0.0001
Word Reading Corectness *%correct*	90	74	33.3	<0.0001
Word Reading Time *seconds*	71.7	170.1	28.8	<0.0001
Pseudoword Reading Correctness %correct	76	52	34.9	<0.0001
Pseudoword Reading Time *seconds*	117.1	218.7	12.6	0.0002
Mottier Test *%correct*	85	65	25.1	<0.0001
Dictation *%correct*	63	51.4	15.2	0.0002
Categorical Perception *Index *(F(1,68))	58.1	36.9	194.7	<0.0001

Table 1 depicts that test performance of the dyslexic children was below test performance of the control children in all measures. Note that for the dyslexic group, the average T-value was 51.4 (ranging from 48 to 54) in the non-verbal intelligence test SPM and 36.9 (ranging from 35 to 38) in the spelling test (T-values were derived from comparisons with age-matched norm-samples). It was required that the DRT test performance was below average (T = 50) and the discrepancy between DRT and SPM performance was at least 10 T-values (1 standard deviation). In German-speaking countries, the diagnosis of dyslexia is based on spelling performance rather than on reading performance. Thus, reading performance was not used as a primary diagnostic criterion. Nevertheless, table 1 depicts, that reading performance was also significantly worse in the dyslexic group than in the control group.

### Stimulation

Three different types of words were presented: 1) HF words, 2) LF words, and 3) PS words. Content words were selected from the German version of the standardized word-database CELEX [32]. HF words were selected to be as high frequent as possible (1091-104 per million words text), LF words were supposed to be as low frequent as possible (1–9 per million words text). All words were nouns. PS words were generated by shuffling letters of actual words so that they were still pronounceable, orthographically legal but non-existing German words. All words and PS words were 5 to 7 letters long, written in capital black letters on a white background.

100 HF, 100 LF and 100 PS words were selected. Together they formed a block of 300 words being presented in a randomised fashion. Each block was presented twice at a presentation rate of 1/350 ms. There was no temporal gap between successively presented words.

Words were generated in bitmap-format; "Presentation" software (Neurobehavioral Systems, Inc.) was used for stimulation. Words were screened onto a white projection field (max. word size: 20–32 cm × 9 cm, 1.4 m away from the subject's eyes) at the ceiling of the chamber using a video beamer (JVC™, DLA-G11E) and a system of mirrors.

Subjects were told that they would see different words and PS words on the screen and were instructed to read them as carefully as possible. They were also told that words might flash so fast that reading would be difficult, but that they should still try as hard as they can. Children were also asked to name some of the words they saw after the experiment.

### MEG recording

MEG was recorded using a 148-channel whole-head magnetometer (MAGNES™ 2500 WH, 4D Neuroimaging, San Diego, USA). Subjects were lying supine in a comfortable position in the magnetically shielded room (Vakuumschmelze Hanau). Data were recorded with an online high-pass filter of 0.1 Hz and a sampling rate of 508.63 Hz (bandwidth 100 Hz), as well as standard noise reduction procedures. Recording was continuous.

For artefact control, eye movements (EOG) were recorded from four electrodes attached to the left and right outer canthus and above and below the right eye, as well as cardiac activity (ECG) via two electrodes, one on each forearm. A Synamps amplifier (NEUROSCAN) served for the recording of EOG and ECG. A video camera installed inside the chamber allowed monitoring the subject's behaviour and compliance at any time throughout the experiment.

### Data analysis

Data were noise-reduced and corrected for cardiac activity. For each subject data epochs with a 350 ms baseline and a post-trigger window of 350 ms were generated. Epochs containing artefacts (signals > 120 μV in the EOG and signals > 5pT in the MEG-channels) were rejected. The remaining epochs were averaged separately for the three word conditions. As the next step, a time-frequency analysis with a complex Gabor wavelet [33] and a f_0_/σ_f _ratio of 7 was computed for all MEG-channels. Power spectra were retrieved from the FBA for each time point and frequencies between 10 and 100 Hz. In order to reduce the amount of information, selected channels above frontal, temporal and occipital regions in both hemispheres were averaged to form 6 channel groups (left and right frontal, left and right temporal, left and right occipital). Frontal and temporal channel groups consisted of 20 channels, occipital channel groups consisted of 15 channels.

**Figure 1:**
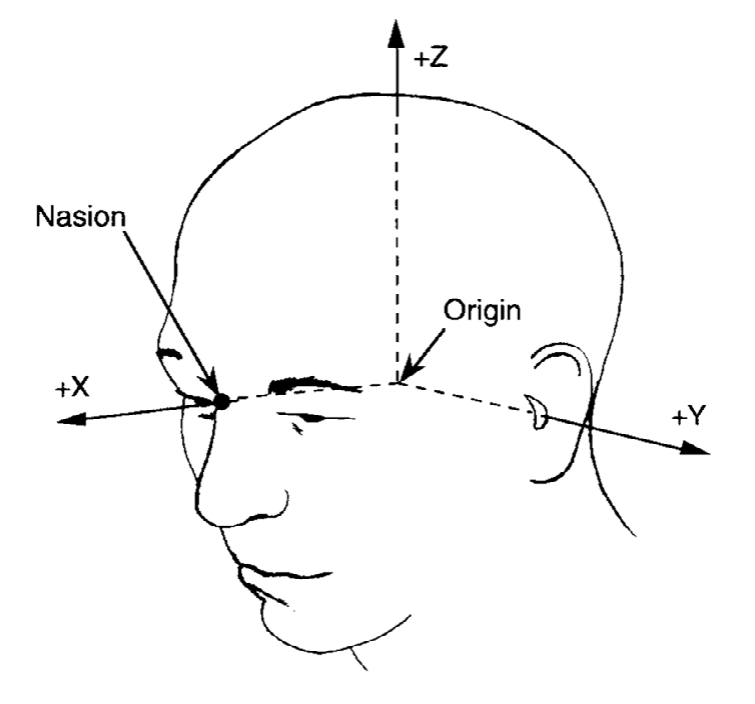
Head coordinate system.

Channel groups were selected individually based on pickup coil positions. The centre and neighbouring pickup coils were estimated as being closest in terms of angle to predefined positions (all positions (x, y, z, see Fig. 1) in cm: left frontal (8, 4, 5), right frontal (8, -4, 5), left-temporal (0, 7, 5), right-temporal (0, 7, 5), left-occipital (-8, 4, 5), right-occipital (-8,-4, 5)). This assured that channels over the same brain regions were averaged for all the subjects.

### Statistical analysis

Since the exact timing of maximum activity, as well as the spectral frequency of activation peaks varies considerably between subjects, it was decided to investigate maximum evoked power amplitudes as dependent variable rather than mean amplitude in a selected time-frequency bin (see results section). Using mean activity in a time-frequency window might have led to blurred results.

Statistical analysis was done with mixed models using the PROC MIXED module of SAS™. Covariance parameters were estimated with the restricted maximum likelihood method (REML). Maximum amplitude of evoked power (MAX) in the time-frequency bin 80–150 ms and 15–35 Hz (see results section) was dependent variable, HEMISPHERE (left, right), WORDCLASS (HF, LF, PS) and GROUP (control, dyslexic) were fixed effects. PATIENT nested in GROUP was used as random factor. Variance structure was *variance components (VC)*. Least square means were estimated with the restricted maximum likelihood method (REML). Tukey-Kramer test was used for post-hoc investigations of significant differences. In cases of significant differences, spectral frequency (Hz) and latency (ms) of the maximum amplitudes were analyzed as dependent variable. Fixed effects and random effect were the same as described above. *Only significant main effects, interactions and post hoc tests are reported*.

Where significant group effects were found, correlations were calculated between the correspondent dependent variable (MAX amplitude, spectral frequency or latency) and performance at the behavioural tests.

## Results

**Figure 2:**
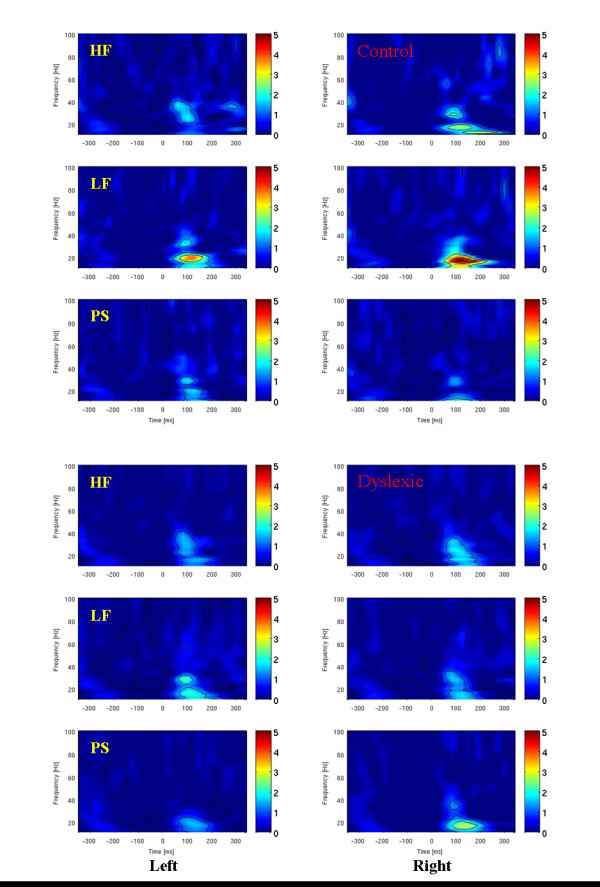
Average evoked power for high, low frequent and pseudo words in both hemispheres. Most activity was found between 80 and 150 ms and between 15 and 35 Hz. The top half of the figure illustrates data of control children, the bottom half data of dyslexic children.

Figure 2 shows the *average power *evoked by the three word conditions for the control children in occipital regions. Evoked power is expressed in z-values. Most activity can be seen between 15 and 35 Hz in a time window from 80 to 150 ms. Thus, further analysis was performed in this time-frequency bin. *MAX amplitudes *were determined and statistically analysed in the time-frequency bin per condition, hemisphere and person.

### Results statistical analysis

#### Frontal and temporal channels

No differences between the groups or interactions between GROUP and WORDCLASS were found for frontal and temporal channels.

#### Occipital channels

##### MAX amplitude

**Figure 3:**
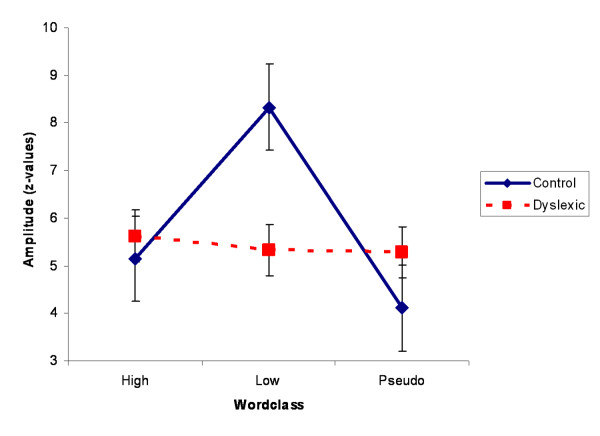
Interaction GROUP*WORDCLASS. LF words were related with higher amplitudes only for the control children.

The interaction GROUP*WORDCLASS (F(2,146) = 4.62, p = 0.01) was found. Figure 3 displays the LS means of GROUP*WORDCLASS. As can be seen, clear amplitude differences between the wordclasses were found for the control children, whereas no such clear differences were apparent for the dyslexic children. Post hoc testing revealed that within the group of control children, LF words led to higher amplitudes than HF words (p = 0.01) and PS words (p = 0.001). The two groups differed in amplitude of LF words. Amplitudes in the group of control children were higher than amplitudes in the group of dyslexic children (p = 0.005).

##### MAX spectral frequency

**Figure 4:**
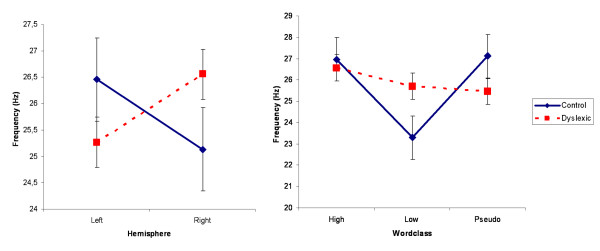
(A)Interaction GROUP*HEMISPHERE. Right hemispheric activity was related with higher spectral frequencies only for dyslexic children. (B)Interaction GROUP*WORDCLASS. LF words were related to lower spectral frequencies only for the control children.

The interaction GROUP*HEMISPHERE (F(1,73) = 4.48, p = 0.038) was found. Whereas MAX frequency was higher in the left hemisphere compared to the right hemisphere in the group of control children, the opposite pattern could be seen for the group of dyslexic children. However, the only statistically significant post-hoc difference was found between left and right hemispheric spectral frequencies for the dyslexic children (p = 0.047, see figure 4).

Finally, the interaction GROUP*WORDCLASS (F(2,146) = 3.07, p = 0.049) was revealed. While MAX frequency differed between the wordclasses within the group of control children, this was not the case for the dyslexic children. In the control group, LF words were associated with lower spectral frequencies than HF (p = 0.01) and PS words (p = 0.009). Furthermore, spectral frequency of LF words was lower for the control children than for dyslexic children (p = 0.04, see figure 4).

**Figure 5:**
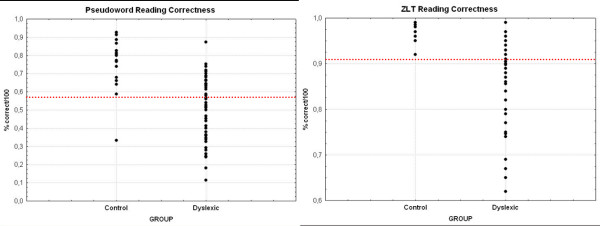
(A) Scatterplot of control children and dyslexic children for pseudoword reading. (B) Scatterplot of control and dyslexic children for ZLT reading performance.

##### Latency of MAX

No significant group differences were found for MAX latency. Latencies for the different word types were 100.2 ms (HF words), 102.3 ms (LF words) and 87.57 ms (PS words).

Correlation between MAX amplitude, frequency and behavioural tests (p < 0.05)

##### MAX amplitude

For **LF words**, a significant correlation was found between test performance in the DRT and MAX amplitude (r = 0.25), as well as between performance in the dictation and MAX amplitude (r = 0.24). I.e. higher spelling ability seemed to be associated with higher MAX amplitudes.

##### MAX spectral frequency

For **HF words**, a linear relationship was found between word reading performance and MAX frequency (r = 0.24). A negative correlation was revealed between word reading time and MAX frequency (r = -0.27). I.e. a small amount of errors and high reading speed at the word reading test was related with higher MAX spectral frequencies.

For **LF words**, negative correlations were found between performance in categorical perception and MAX spectral frequency (r = -0.30), performance in the Mottier test (r = -0.37) and MAX spectral frequency as well as between performance in the SPM and MAX spectral frequency (r = -0.35). I.e. both, good performance in tests of phonological awareness and good performance at the nonverbal IQ test were associated with a lower MAX spectral frequency.

##### Subcategorisation of the dyslexic group

We categorised the group of dyslexic children based on their performance in the pseudoword reading test and their reading correctness in the ZLT. In the case of pseudoword reading, children who scored lower than 58% correct were classified as "poor" (n = 33), the rest as "good" (n = 22, see fig. 5). In case of ZLT reading correctness, dyslexic children were classified as "poor" (n = 34) when they performed worse than 92% correct, the rest was classified "good" (n = 21, see fig. 5).

**Figure 6:**
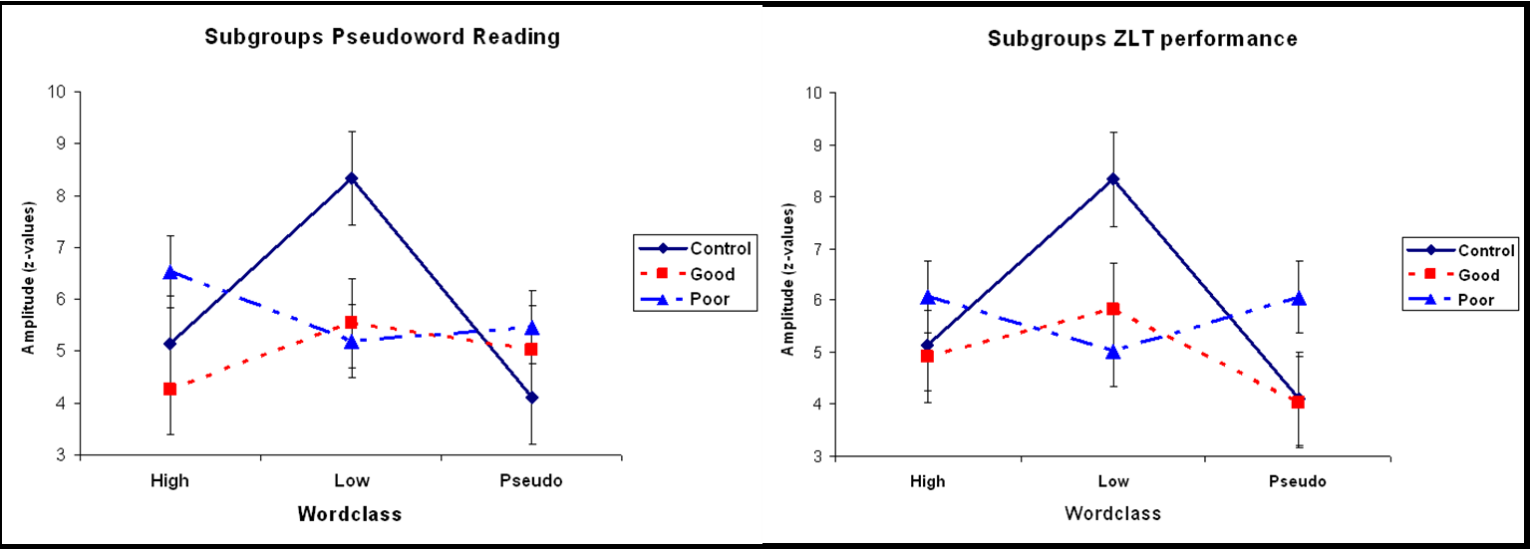
(A) Activation pattern of control children, good and poor pseudoword readers (left side) as well as control children, good and poor readers (right side).

In both cases of subcategorisation, the significant interaction GROUP*WORDCLASS was found (F(4,144) = 9.09, p = 0.02 for the pseudoword subgroups and F(4,144) = 3.18, p = 0.02 for the ZLT subgroups). As can be seen in figure 6, the activation pattern of children who were classified as good readers resembled the control group more than the activation pattern of poor readers. The only statistically significant post-hoc differences were found for the control group, however (see above).

## Discussion

While control children displayed a typical pattern of higher activity following LF words than HF words, this word frequency effect was not present for dyslexic children. A word frequency might reflect different strategies reading HF and LF words. HF words are possibly recognised as a unitary pattern [18], whereas LF words have to be phonologically assembled from sublexical parts following grapheme-to-phoneme correspondences [11]. The latter procedure is likely to draw more processing resources and thus results in higher amplitudes in the EEG or MEG signal [17]. Our finding of a word frequency effect in healthy control children is in line with several studies on visual word processing (e.g. [14-17]). The absence of a word frequency effect in dyslexic children might result from a specific difficulty encoding LF words. As formulated in our hypotheses, control and dyslexic children did not differ processing HF words. We assume this might be the case, since dyslexics are generally not impaired reading highly familiar words. Neither did the groups differ processing PS words. One possible explanation is that processing (in terms of wordform recognition) succeeds for HF words in both groups and LF words in the control group (with more effort due to phonological assembly) but not for PS words due to lacking word representation. Similar amplitudes of HF and PS words thus might reflect different underlying processes. It is imaginable that highly tuned neural cell assemblies respond to the visual pattern of HF words resulting in a relatively low amplitude [17], whereas a similarly low amplitude for PS words may result from a lacking wordform representation for PS words.

Dyslexic children performed significantly poorer than control children in the behavioural pseudoword reading test while no group difference was present in either evoked power or frequency following visual pseudoword stimulation. This finding seems unexpected. However, it should again be pointed out that we used a relatively high presentation rate, because we were interested in *automatic *reading processes rather than in reading *performance*. Using the presentation rate of 1/350 ms processing of one word later than 350 ms post stimulus is at least partly suppressed, because the next word is already present. Due to the limited processing time we assume that pseudowords "drop out" of the processing cascade once it is detected that no real word is present. This seems to happen for both dyslexic and control children.

In an fMRI study, Mechelli and colleagues [34] found words and pseudowords to activate bilateral occipital cortices as well as left posterior inferior temporal regions. Interestingly, they did not find any areas that were activated more by words than by pseudowords, apart from the left middle occipital gyrus. This, however, was an effect of an activation *decrease *for pseudowords rather than an activation *increase *for real words. Word frequency was not controlled in this study, so that no inferences can be made about low and high frequency words. If we averaged low and high frequent words in the present study, we would also find higher amplitudes for real words than for pseudowords (in the control group, see fig. 3). Thus, our data are in line with the results of Mechelli and colleagues.

We had expected group differences for LF words. Reduced amplitudes in the dyslexic children are possibly a consequence of their reduced ability to read via the graphophonological route which in turn might result from reduced phonological awareness. Whereas control children manage to successfully apply grapheme-phoneme matching, dyslexic children might not be able to do so resulting in lower amplitudes for LF words compared to control children. This view is supported by the results derived from subgrouping the children into good and poor readers. Both, children who were "good" readers of pseudowords and who performed well in the ZLT resembled control children in their activiation pattern more than dyslexic children who were poor readers. Interestingly, we also found significant correlations between MAX amplitude for LF words and performance in the dictation as well as the standardised spelling test. This supports the idea that good spelling performance is related to the ability to apply grapheme-phoneme correspondences (reflected in higher amplitudes for LF words).

One might argue that dyslexic children were simply not able to read the words at a presentation speed of 1 word per 350 ms. If that was the case, we would have expected processing differences between the groups for all three wordtypes. However, the groups differed solely in their brain responses to LF words. It should also be noted that we were not interested in *reading performance *at a relatively high presentation rate, but in *automatic reading processes *triggered by a rapid serial visual stimulation with different wordtypes. Under this premise, we interpret that the graphophonological reading route is automatically activated for words that are not represented as unitary objects in control children, whereas this process is not activated in dyslexic children. It should also be noted that reading and even retrieval of *word meaning *is possible at much higher presentation rates (1 word per 54 ms, [24]) using RSVP.

In the present study, interactions between GROUP and WORDCLASS occurred at occipital channels. The occipital cortex has been repeatedly found to be relevant for linguistic processing. Using fMRI, Polk and colleagues [35] found a left occipitotemporal region being more sensitive to letters than to digits. In another fMRI study, Bokde et al. [36] revealed functional connectivity between left inferior frontal and occipital areas only for words, pseudowords and letter strings, but not for false font strings. Patients with lesions to the left temporo-occipital cortex have difficulties reading and spelling comparable to dyslexic symptoms. These patients are especially impaired at spelling irregular and LF words [37]. It has been claimed that extrastriate regions in the left hemisphere might be crucial in the acquisition of orthographic word representations [38].

Assadollahi & Pulvermüller [16] found their word frequency effect at left occipitotemporal regions. The authors state that this region might correspond to the *visual word form area *(VWFA, [39]), which has – although not undisputed [40] – generally been found to be activated stronger by visual words and pseudowords than by other visual stimuli (see [41]). The VWFA seems to be modality-specific, insensitive to semantic modulation [42] and can be activated without awareness. Interestingly, it has been shown that dyslexic adults activate the VWFA less than controls in response to visual words and pseudowords [43-46]. It thus appears that left occipitotemporal regions are crucial for fluent and automatised word recognition.

Jobard et al., [12] performed a metaanalysis of 35 neuroimaging studies on reading and found an activation cluster around the occipitotemporal sulcus thus supporting the existence of a VWFA. The authors conclude that prelexical processing of words and pseudowords might take place in this area, i.e. segmentation, classification and the relay of visual word information to other cortical regions for further analysis. This view is supported by Coultheart and Rastle [11], who already stated in the original description of their reading model that initial processing stages are shared between the two reading routes (direct and graphophonological).

From our results of course, we cannot claim to show activation of the VWFA, since our channel selection covered more cortical areas than the VWFA. Neither did we find left lateralised results. Nevertheless, it is likely, that VWFA activation is strongly *contained *in our effects, especially since the effects were found rather early (~100 ms) – probably reflecting prelexical processing. If Jobard et al's [12] view is correct that occipitotemporal regions might be involved in prelexical processing and the relay of visual word information to other cortical areas, it might be assumed that there is a specific deficit in dyslexic children concerning LF words. Control children showed stronger activation for LF words compared to HF or PS words (see above). Dyslexic children did not. LF words can only be successfully decoded applying grapheme-phoneme correspondences – the reading requisite where dyslexics seem to be most impaired.

The word frequency effect for amplitudes was accompanied by a word frequency effect for spectral frequencies. LF words were related to lower spectral frequencies (~23 Hz) than HF and PS words (~27 Hz) only in control children. Maximal activity for LF words thus peaked in a high beta-band range, while HF and PS words peaked in a low gamma-band range. Xiang et al., [47] also investigated neuromagnetic spectral distribution during word and non-word stimulation and found frequency changes between 15 to 30 Hz to be crucial for word and non-word processing at occipital sites. The authors related these frequency changes to spatiovisual information processing. They interpreted that implicit word processing is automatically activated as soon as words are present in the visual field, even if reading is not intended. Another study on power changes during various cognitive tasks was performed by Fitzgibbon et al. [48]. They reported an increase in gamma activity in the posterior cortex especially during reading.

Wrobel [49] found increased beta activity (15–25 Hz) during visual attention in primary and higher order visual areas in EEG experiments. The author proposes that beta band activity might have the general role of an attention carrier comparable to the role of alpha activity in idle arousal, or gamma activity in feature integration processes. Support for the meaning of beta activity in attention also comes from the field of attentional disorders. It has generally been found that children with attentional deficit hyperactivity disorder (ADHD) show decreased levels of beta activity in posterior regions compared to healthy control children (for review see [50]). Finally, Gross et al., [51] also reported beta activity to play an important role in attentional processes by mediating interactions of a widely distributed attentional network. The authors argue that changes in synchronisation might reflect changes in attentional demand of a task. In this view, we might interpret selective beta band activity for LF words in control children to stem from an increase in attention. It is possible that unfamiliar LF words draw more attention than highly familiar HF words or pseudowords (that do not have any ascribed meaning) in skilled readers. Although speculative, it might be assumed that an increased attentional level is necessary for the more demanding processing of LF words. It appears as if the whole cascade of processing steps necessary for decoding LF words is dysfunctional in dyslexic children.

In the present study, significant negative correlations were revealed between the spectral frequency of LF words and performance in the SPM, Mottier test as well as categorical perception. The latter two correlations are of particular relevance, since both Mottier test performance and categorical perception ability reflect phonological awareness. In the Mottier test, children are only required to repeat back pseudowords the experimenter reads out to them. I.e. the ability is measured, if children perceive phonemes correctly. In the test of categorical perception, the children had to categorise if a syllable sounds more like /ba/ or /da/ (when the formant transition period of the syllable is varied on a ten-item continuum with 1 representing a clear /ba/ and 10 representing a clear /da/). Thus, this test is also a measure of phonological awareness. Dyslexic children performed worse in both tests, i.e. they made more errors repeating back pseudowords and were less certain if they heard /ba/ or /da/. They also did not show lower spectral frequencies for LF words compared to HF and pseudowords as control children did. Thus it appears that there is a relationship between phonological awareness and spectral frequencies for LF words. Lower spectral frequency for LF words seems to correspond to higher phonological awareness. Additionally, high spectral frequency values for HF words were correlated with good reading performance and short reading time. Interestingly, there were no correlations between spectral frequency and PS words. This might also strengthen the view that the seemingly similar processing of HF and pseudowords are different in nature.

Besides the lacking word frequency effect in dyslexic children, the interaction GROUP*HEMISPHERE was revealed in the present study. In control children, left occipital activity peaked at 26.5 Hz and right occipital activity peaked at 25 Hz. This was exactly reversed for the dyslexic children. Nevertheless, the meaning of this interaction is hard to interpret, since the differences in spectral frequency are very small (1.5 Hz). It is likely that the reversed pattern in dyslexia is related to a functional meaning. Our exact knowledge about single frequencies so close together is unfirm, however.

## Conclusion

The aim of this study was to investigate brain responses triggered by different wordclasses in dyslexic and control children. For this purpose we utilised a RSVP design and performed wavelet analysis on the evoked activity. We had hypothesised that dyslexic children should mainly differ from controls processing LF words due to their lacking ability to read via the graphophonological route. This was confirmed at the level of evoked power amplitude and its corresponding spectral frequency. However, the lacking differences between word types in the dyslexic group raise the question if dyslexic children were able to process the words presented in rapid serial fashion in an adequate way. Therefore the present results should only be interpreted as evidence for a specific sublexical processing deficit with caution.

## Competing interests

The author(s) declare that they have no competing interests.

## Authors' contributions

Isabella Paul: Conceptualisation and conduction of the study, data analysis, writing of the manuscript.

Christof Bott: Conduction of the study, analysis of behavioural data.

Christian Wienbruch: Programming of analysis software, data analysis, proof reading of manuscript.

Thomas Elbert: Conceptualisation of the study.

## Pre-publication history

The pre-publication history for this paper can be accessed here:


